# A New Anorganic Equine Bone Substitute for Oral Surgery: Structural Characterization and Regenerative Potential

**DOI:** 10.3390/ma15031031

**Published:** 2022-01-28

**Authors:** Alessandro Addis, Elena Canciani, Marino Campagnol, Matteo Colombo, Christian Frigerio, Daniele Recupero, Claudia Dellavia, Marco Morroni

**Affiliations:** 1 CRABCC Animal Lab, Biotechnology Research Center for Cardiothoracic Applications, Rivolta d’Adda, 26027 Cremona, Italy; alessandro.addis@crabcc.com (A.A.); marino.campagnol@unimi.it (M.C.); 2 Department of Biomedical Surgical and Dental Sciences, Università degli Studi di Milano, 20122 Milan, Italy; elena.canciani@unimi.it (E.C.); claudia.dellavia@unimi.it (C.D.); 3 Bioteck S.p.A., Arcugnano, 36057 Vicenza, Italy; m.colombo@bioteck.com (M.C.); c.frigerio@bioteck.biz (C.F.); d.recupero@bioteck.biz (D.R.)

**Keywords:** equine bone substitute, bone formation, xenograft, anorganic bone

## Abstract

Different xenogeneic inorganic bone substitutes are currently used as bone grafting materials in oral and maxillo-facial surgery. The aim of the present study was to determine the physicochemical properties and the in vivo performance of an anorganic equine bone (AEB) substitute. AEB is manufactured by applying a process involving heating at >300 °C with the aim of removing all the antigens and the organic components. AEB was structurally characterized by scanning electron microscopy (SEM), X-ray diffraction (XRD), X-ray fluorescence (XRF), and Fourier-transformed infrared (FT-IR) spectroscopy and compared to the anorganic bovine bone (ABB). In order to provide a preliminary evaluation of the in vivo performance of AEB, 18 bone defects were prepared and grafted with AEB (nine sites), or ABB (nine sites) used as a control, in nine Yucatan Minipigs. De novo bone formation, residual bone substitute, as well as local inflammatory and tissue effects were histologically evaluated at 30 and 90 days after implantation. The structural characterization showed that the surface morphology, particle size, chemical composition, and crystalline structure of AEB were similar to cancellous human bone. The histological examination of AEB showed a comparable pattern of newly formed bone and residual biomaterial to that of ABB. Overall, the structural data and pre-clinical evidence reported in the present study suggests that AEB can be effectively used as bone grafting material in oral surgery procedures.

## 1. Introduction

Presently, bone grafting is a major treatment modality in oral surgery for bone volume preservation as well as for augmentation procedures [1].

An ideal bone graft should foster natural healing through osteoconductive, osteoinductive, and osteogenic mechanisms, be biocompatible, and not evoke any inflammatory response. In addition, it should be sterilizable and readily available at a reasonable cost [2]. It has been widely described in the literature that materials that feature slow resorption kinetics, without disturbing the natural bone remodeling process occurring around them, are able to obtain positive clinical results and a long-term volume stability [3,4]. Morphology, particle size, and chemical composition of a bone graft material (as largely determined by its production process) significantly influence its resorption rate [4]. Therefore, a better and more comprehensive understanding of its physicochemical properties seems necessary to achieve predictable biological and clinical response. Among the different available augmentation materials, autologous bone still represents the gold standard for bone regeneration [2,5] but has several disadvantages, including limited availability, the need for an additional surgery, and potential donor site morbidity [6,7]. Its limitations have stimulated the search for alternative solutions, and currently many alternative bone substitutes, either natural or synthetic, are available in clinical practice [8]. Among natural materials, xenogeneic bone grafts (derived from mammals’ species) are the most common bone substitutes utilized clinically, due to their ready availability and their similarity to human bone regarding chemical composition and structure [9]. Indeed, among mammals, bovine bone was the first considered due to its high availability. To avoid unwanted immunological reactions and any risk of cross-infections, xenogeneic bone grafts undergo specific treatments of deantigenation and sterilization [7,8]. 

Among these processes, thermal treatment is one of the most used, and it is often applied on bovine bone, but also on porcine bone [10,11,12]. The elevated temperature applied (between 300 and 1200 °C depending on the different processes) [13] neutralizes the antigenic components while maintaining the natural architecture of the bone [7,8]. The output is an anorganic bone hydroxyapatite with physicochemical properties favorably associated with bone repairing osteogenesis and osseous growth [14,15,16,17,18]. 

One of the best-characterized xenogeneic bone substitutes obtained through thermal treatment is anorganic bovine bone (ABB). Indeed, ABB is characterized by a macro- and micro-porous structure similar to human cancellous bone [19,20], which serves as physical scaffold for the migration of bone-forming cells and provides an optimal microenvironment for bone ingrowth [21]. It is resorbed slowly, supporting the process of natural bone remodeling around it [15,22,23]. Clinically, its biocompatibility, stability, and long-term efficacy have been widely demonstrated in most varied indications, including ridge preservation, bone augmentation, and periodontal regeneration [14,15,16,17,18,24].

As with bovine bone, equine bone displays high similarity with human bone [25,26]. Moreover, equine bone has some additional advantages such as the absence of ethical issues and the intrinsic stability of its proteins that exclude the equine from the prion transmitting species as stated by the European Commission Regulation N. 722/2012 of 8 August 2012 [27]. Recently, an anorganic equine bone (AEB) that is subject to similar manufacturing process has been introduced in the market. AEB is produced by treating the cancellous equine bone with a very high temperature to eliminate all the organic components (including bone collagen) and to achieve a controlled decarbonation of the apatite crystals. So far, no studies investigating the chemical and biological properties of this novel biomaterial have been published. 

The aim of the present study was to assess the physicochemical and structural properties of AEB, as well as its in vivo performance in an animal model of mandibular bony defects.

## 2. Materials and Methods

### 2.1. Materials

Anorganic equine bone (AEB, Calcitos^®^—Bioteck S.p.A., Arcugnano, Vicenza, Italy) particles are derived from cancellous bone of equine origin. The product is sterilized by beta-irradiation at 25 kGy. 

Anorganic bovine bone (ABB, Bio-Oss^®^—Geistlich Pharma AG, Wolhusen, Switzerland) is bovine-derived and its sterilization takes place with the application of gamma-irradiation [22]. Both AEB and ABB particles are obtained through a proprietary extraction process that involves treatment with strong alkalis and solvents under high-temperature processing of >350 °C [13]. 

Because of the large number of publications describing the properties and the clinical performances of ABB, it has been used as benchmark in the different test performed in the present study.

### 2.2. Physicochemical and Morphological Characterization

Morphological characterization and measurement of the particle size of the materials were carried out through scanning electron microscopy (SEM; Phenom XL, Phenom-World, Eindhoven, The Netherlands) operating at a range of 4.8 kV to 20.5 kV or at 5 kV of electron acceleration, respectively. The sample was placed in a holder composed of conductive carbon and golden surface, and the image was obtained by backscattering radiation. Morphological analysis was performed using Scanning Electron Microscope XL Phenom combined with software 3D Roughness Reconstruction (Phenom-World, Eindhoven, The Netherlands). The diameters of the particles acquired from each SEM image were measured using the embedded image analysis software (Phenom Pro Suite/Fibermetric). Every particle was measured using the approach of the medium diameter, intended as the dimension visually equivalent at the diameter of the particle if its visible surface is a circle. To ensure the representativeness of the particle distribution, the counting was done on all the particles attached, during the preparation procedures, on two different conductive carbon supports, each one with a diameter of 20 mm. The number of particles on each filter is random and, for this sample type, not directly connected with the particle size. 

X-ray diffraction experiments were performed in order to identify the crystalline phases in the xenografts by using ARL X’TRA X-ray diffraction (Thermo Fisher Scientific, Waltham, USA) with Cu–Kα ray (45 kV, 40 mA). Spectra were recorded in the 2θ range of 4°–60° at a step size of 0.010° and a step time of 0.40 min.

An X-ray fluorescence (XRF) spectrometer (SPECTRO XEPOS 3, AMETEK, Berwyn, Germany) was used to quantify the elemental chemical composition of the biomaterials. The data were acquired with an axial wavelength dispersive XRF unit.

Both ABB and AEB were analyzed via infrared spectroscopy using a Cary 630 FT-IR (Agilent Technologies, Santa Clara, CA, USA) instrument with an ATR module. Spectrum window was collected from 800 to 3800 cm^−1^ with a resolution of 2 cm^−1^. A qualitative analysis of the spectra was then performed by comparing the wavenumber of the most significant peaks with that of a reference wavenumber library, in order to identify the main functional molecular groups.

### 2.3. Animals

Nine adult (20–24 months) Yucatan Minipigs, belonging to the same progeny, were used in the study. Animal experimentation was conducted in compliance with ISO 10993-2, European Directive 2010/63 EU, and D.Lg 26/2014, the Italian Law on the protection of animals used for scientific purposes.

### 2.4. Study Protocol and Randomization

The present study was designed as a randomized-controlled experimental study. In order to provide a preliminary evaluation of the performance of AEB and ABB, a total of 18 mandibular bone defects were prepared and grafted with AEB (nine sites), or ABB (nine sites) in nine Yucatan Minipigs. Each animal provided two grafting sites; one site was grafted with ABB, the other with AEB. Animals were divided into two groups to allow the subsequent evaluation of device resorption time, amount of newly formed bone, and local inflammation effects at two different time-points (30 and 90 days after surgery). At each time-point, animals were sacrificed (four animals at 30 days and five animals at 90 days), and bioptic samples at each grafting site were collected and histologically evaluated.

### 2.5. Grafting Surgical Procedure

The surgical procedure was performed under sterile conditions. General anesthesia was induced by intramuscular injection of ketamine (10 mg/kg) and midazolam (0.5 mg/kg), followed by administration of an oxygen and isoflurane mixture through a mechanical respirator. Anesthesia was maintained with a mixture of isoflurane 3.5% and oxygen 100%.

Through subangular incisions, the lateral portion of the mandibular body and ramus were exposed enough to allow the preparation of two standardized intraosseous defects. Defects measuring 5 mm in diameter and 5 mm in depth were prepared using a trephine with copious saline irrigation. Each defect was filled with AEB or ABB and covered with a pericardium membrane (Heart^®^, Bioteck S.p.A., Arcugnano, Vicenza, Italy). Finally, surgical sites were closed in multiple layers using a resorbable suture. Each step of the surgical procedure was documented by a complete set of images (Supplemental Figure S1).

All the animals were constantly monitored for 72 h after the surgery in order to assess their correct physiological recovery.

### 2.6. Sampling and Histological Preparation

Animals were sacrificed by intra-venous administration of potassium chloride saturated solution, and the samples were collected at 30 and 90 days after surgery. Biopsies of each grafting site were harvested using a 10 × 4 mm diameter trephine bur, placing the trephine at the center of the defect and collecting a bone core including basal bone and the grafting tissue for the entire depth of the defect. Each sample was fixed in buffered 10% formalin, decalcified by Osteodec (Bio Optica, Milano, Italy), dehydrated in ascending alcohol scale infiltrated, and finally embedded in paraffin (Bio-Plast, Bio Optica, Milano, Italy). Three serial longitudinal sections of 6 μm were obtained in the central portion of the block with a microtome (Leica Biosystems, Milano, Italy) and stained with Carazzi’s Hematoxylin and Eosin in order to perform morphological and histomorphometric analysis.

Images of the samples were captured using high-resolution digital scanner Aperio CS2 (Leica Biosystems, Milano, Italy) and analyzed with Image Scope software (Leica Biosystems, Milano, Italy).

### 2.7. Histological Measurements

On each section, a counting grid was used to evaluate the intersection points that fall down on each kind of tissue (regenerated bone, biomaterial, and soft tissue) using the software ImageScope (Leica Biosystems, Milano, Italy). The volume fractions percentage was obtained by the ratio between the intersection points that fall down on each type of tissue and the total intersection points. Implant sites were examined for cell type/response in terms of typing of inflammatory cells and inflammatory infiltrate, in the grafted area (polymorphonuclear cells, lymphocytes, macrophages, plasma cells, giant cells, and necrosis), and tissue response observing neovascularization, fibrosis, and fatty infiltrate in the grafted area. In brief, following ISO 10993-6:2007, Annex E, an experienced pathologist examined 10 photos for each slide at a magnification of 400× in order to evaluate all parameters by using an objective score system in the microscopic field. In each section, a global score between 0 (absence of cells/absence of tissues response) and 4 (packed cells/extensive presence of tissue response) was given to each parameter at all time-points, and the mean value for each sample was given [28].

### 2.8. Statistical Analysis of Cellular Content of Histological Samples

Quantitative variables were summarized as mean ± standard errors for each biomaterial at every time point, considering all histomorphometrical parameters. The characteristics were compared at every time point between biomaterials with the Mann–Whitney test for paired data and then a non-parametric longitudinal analysis was processed using the nparLD R package [29]. The p-values were adjusted for multiple comparisons with the Bonferroni method. All p-values were 2-tailed, with statistical significance set at <0.05. Analyses were performed using R software (version 3.3.2) for Windows.

## 3. Results

### 3.1. Physicochemical and Morphological Characterization of AEB

**Figure 1:**
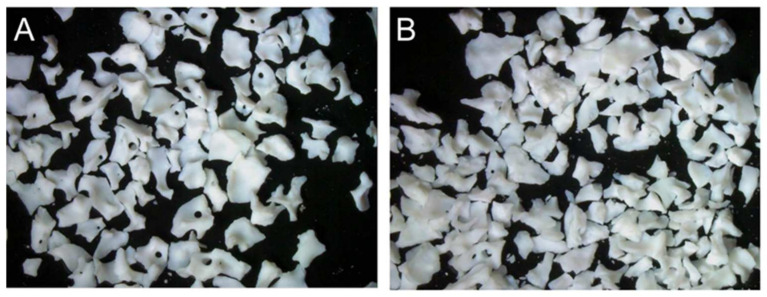
AEB (**A**) and ABB (**B**) particles observed with a stereo microscope.

**Figure 2:**
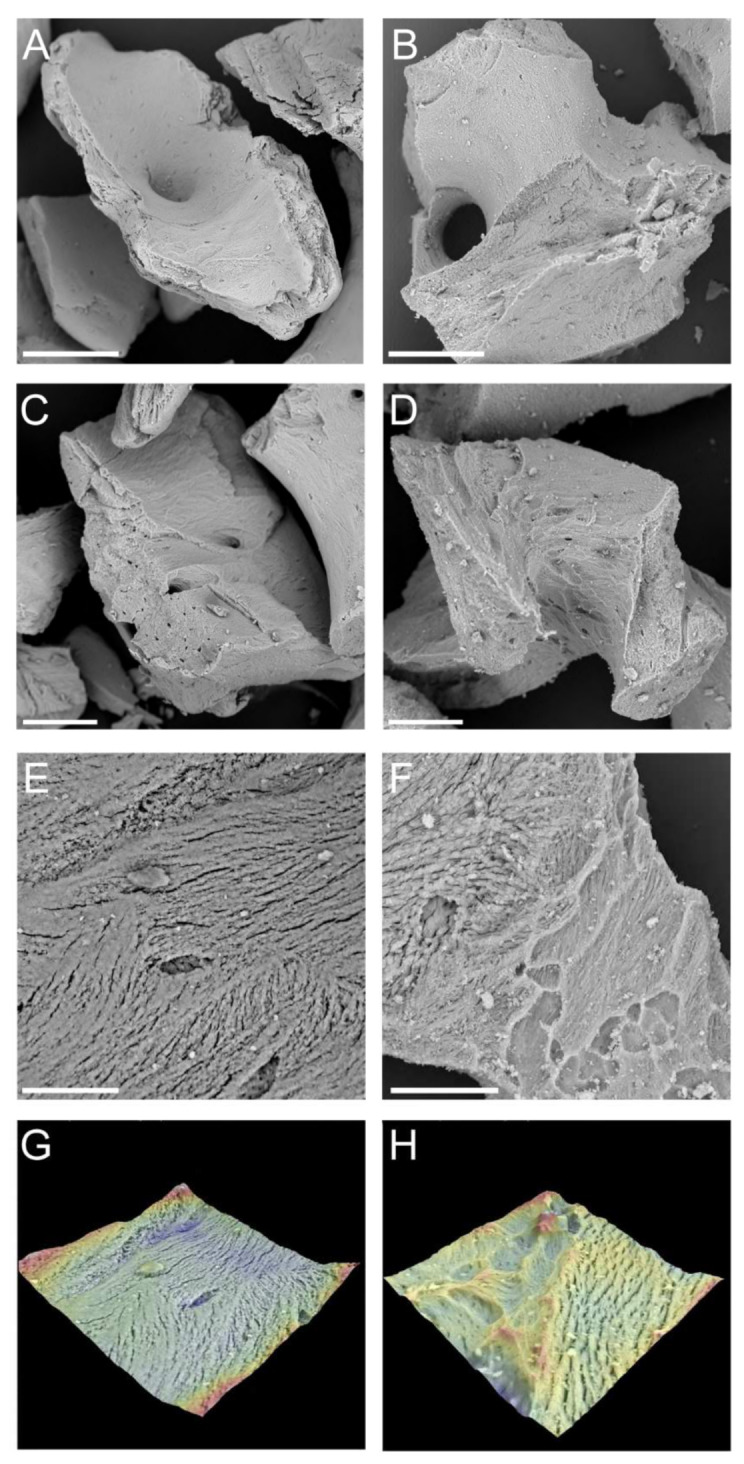
SEM images (**A**–**F**) and 3D surface reconstruction (**G**,**H**) of AEB (**A**,**C**,**E**,**G**) and ABB (**B**,**D**,**F**,**H**). Scale bars: (**A**,**B**): 200 μm; (**C**,**D**): 100 μm; (**E**,**F**): 30 μm.

Optical microscopy observations showed that AEB consisted of particles with similar shape, having some portions rounded and others derived by fragmentation. Some particles had circular holes of variable size (Figure 1). The SEM analysis of AEB showed that the particles had irregular shape and fragmented surfaces (Figure 2A,B). At higher magnifications, bone surfaces presented areas with generally visible stratification, with some inner portions appearing more compact and apparently without stratification (Figure 2C–F). ABB was characterized by a regular arrangement of fibers of the same height, and vacuoles of different diameters but comparable depths (Figure 2H,J). AEB showed a less regular fibrous pattern, with fibers of variable size and orientation, but equal height (Figure 2G,I). Vacuoles were also observed on AEB particles.

**Figure 3:**
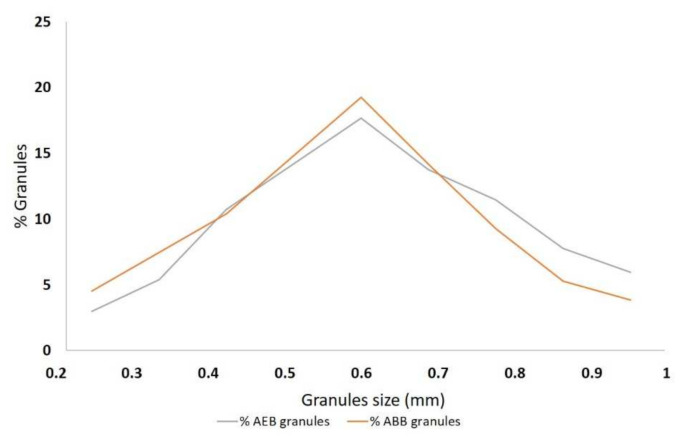
Graphical representation of the granule size distribution of AEB and ABB measured with SEM. On the *x*-axis the granules size in shown in millimeters, whereas on *y*-axis is shown the percentage of granules for each size.

For particles size analysis, a total of 777 and 1240 particles were analyzed for AEB and ABB, respectively. The prevalent particle size of AEB was 0.60 mm (range, 0.120–1.64 mm), which was similar to that of ABB (prevalent diameter, 0.58 mm; range, 0.11–1.54 mm) (Figure 3). The 90% of particle size of both AEB and ABB was between 0.2 mm and 1.0 mm.

**Figure 4:**
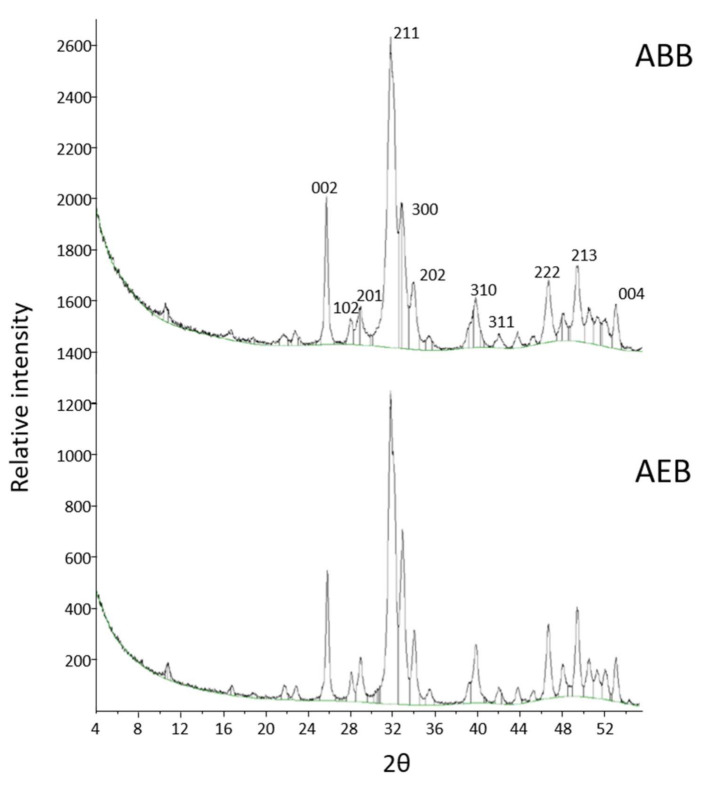
The XRD spectra obtained for ABB (**top**) and AEB (**bottom**). Both biomaterials show the typical peaks of hydroxyapatite.

X-ray diffractometry was qualitatively used to compare the crystalline structure of the biomaterials. The XRD spectra of AEB and ABB were almost superimposable and they showed the characteristics peaks of hydroxyapatite three-dimensional structure (Figure 4).

The determination of elements constituting the bone grafts was then carried out by X-ray fluorescence elemental analysis of the particles. The XRF analysis showed no substantial differences in the chemical composition of the two samples (Table 1). In both biomaterials, the most abundant elements were calcium and phosphorus (Table 1).

**Figure 5:**
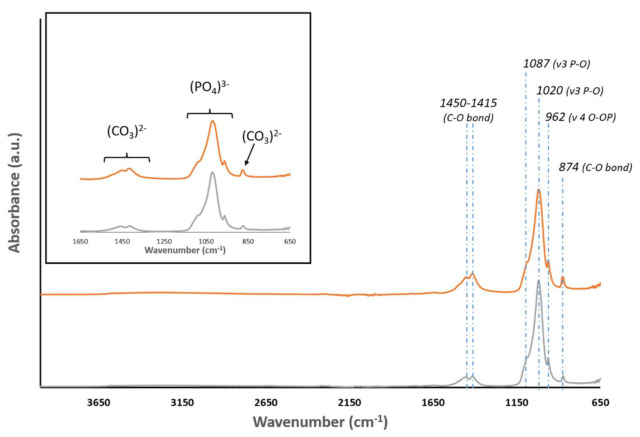
FTIR spectra of ABB (orange) and AEB (gray). Samples of both biomaterials exhibit main peaks around 1450–1415, 1020, 962, and 874 cm^−1^.

Sample characterization was complemented by Fourier transform infrared spectroscopy. Figure 5 presents the FT-IR spectra. Symmetric vibration bending or stretching of the absorption bands can be observed for the C-O bond at wavenumbers of 1450, 1415, and 874 cm^−1^, which can be attributed to CO_3_^2−^ (carbonate ions type B). The band at 962 cm^−1^ is part of the symmetric and asymmetric deformation modes of ν 4 O-OP, whereas the absorption bands in the range of 1020–1087 cm^−1^ correspond to the ν 3 P-O [30,31,32]. 

### 3.2. Resorption, New Bone Formation, and Local Effects after Implantation of AEB and ABB in an Animal Model

In order to evaluate the performance of the two biomaterials, a total amount of 18 bone defects were prepared and grafted with AEB (nine sites), or ABB (nine sites). Device resorption time, amount of newly formed bone, and local inflammation effects were evaluated at 30 and 90 days after surgery.

**Figure 6:**
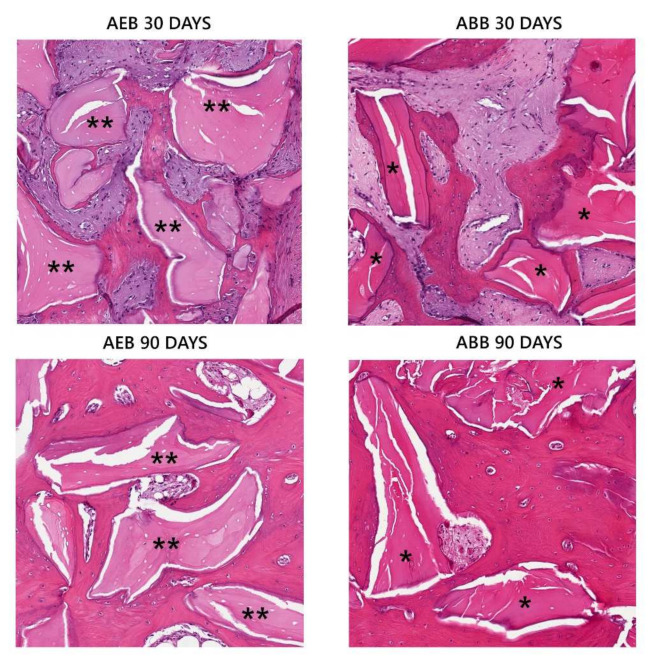
Histological examination of grafted particles at 30 and 90 days from regenerative procedure. At 30 days AEB (**) and ABB (*) particles were surrounded by a thin layer of newly formed bone. At 90 days AEB (**) and ABB (*) particles appeared integrated in extended bony islands.

At the histological examination, with both types of grafts, the biomaterial granules appeared surrounded by a considerable quantity of mineralized matrix at different stages of mineralization (Figure 6). At 30 days, both ABB and AEB particles seemed to be included in a thin layer of osteoid or woven bone (Figure 6). At 90 days several areas of regenerated lamellar bone appeared in both groups (Figure 6), even if a large variability was found between specimens, thus indicating a still ongoing remodeling/regeneration process. In all sites the grafted particles were still present after 90 days from application.

**Figure 7:**
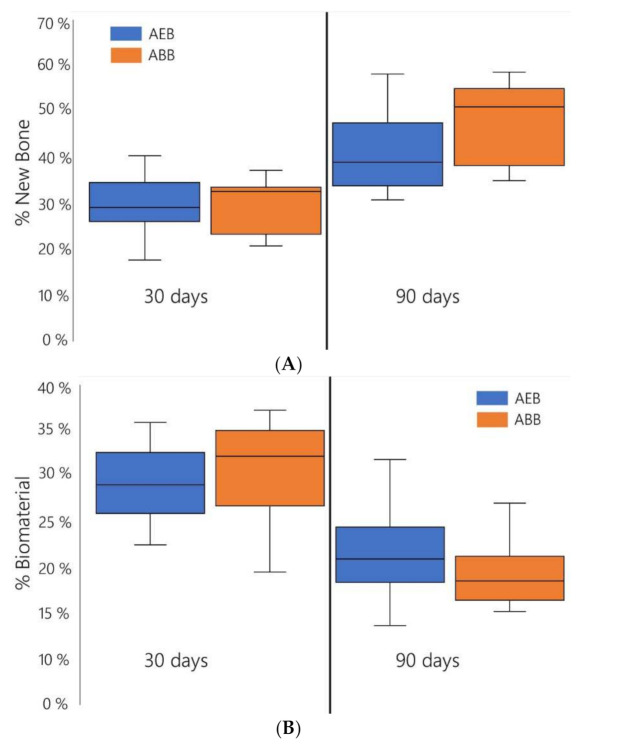
Box plot representation of the percentage of newly formed bone ((**A**) top panel) and of residual biomaterial ((**B**) bottom panel) in samples that were grafted with AEB or with ABB. No statistical differences are present between the two biomaterials.

In both groups the regenerated bone significantly increased over time from 30 to 90 days after surgery (*p* < 0.001) with no significant differences (*p* > 0.05) between ABB and AEB for all time points considered (Figure 7A, top). When considering the residual particles, the percentage of remnants decreased significantly with time (Figure 7B; *p* < 0.0001); a similar trend was found in the two groups. No statistical differences in the percentage of residual particles between groups were observed at the time points considered (Figure 7B; *p* > 0.05).

The assessment of the host response after implantation of biomaterials showed similar local effects in the two experimental groups. In all sites a small inflammatory infiltrate was observed, especially at 30 days, and then diminished at the second time-point (Table 2). The infiltrate was mainly characterized by rare polymorphonuclear cells and rare lymphocytes. Only in some specimens a few plasma cells and macrophages were detected, and no giant cells were seen. At each time point, no significant difference was observed in the number of inflammatory cells in samples grafted with either ABB or AEB (Table 2). Also, no necrotic areas were observed. The tissue response demonstrated a complete absence of fatty infiltrate in all sites of both groups (Table 3). A fibrotic reaction consisting of a moderately thick band was detected in two sites at 30 days, one in ABB and one in AEB.

Considering the neovascularization, both groups showed a minimal proliferation with focal buds of capillaries, and only in a few cases larger vessels with supporting fibroblastic structures were observed, with no statistical differences between groups.

## 4. Discussion

Bone availability is the main prerequisite for safe and predictable outcomes in oral-maxillofacial procedures. However, it can be hindered by bone resorption, which is commonly observed following tooth extraction or tooth loss or can be the result of trauma, pathologies, inflammatory conditions, or chronic/acute infections [33]. Bone grafting may therefore be required in order to either preserve or achieve adequate bone levels. Among xenogeneic bone grafts, mammal species are becoming more and more relevant in regenerative dentistry. They are highly osteoconductive as the three-dimensional structure is similar to that of the human bone and present the advantages that are readily available at reduced costs, are easy to handle, and are slowly resorbed and replaced by the patient’s bone [5,6]. In order to eliminate immunological problems, the bone origin tissue is made non-antigenic using different kind of treatments, namely chemical, enzyme-based, or thermal. Depending on the deantigenation method employed, the resulting bone substitute has different physicochemical and biological features. Whereas the chemical and enzyme-based deantigenation methods generate collagenic bone substitutes showing a high ratio of remodeling with the patient’s bone [34,35], the bone substitutes obtained with a thermal treatment (commonly known as anorganic bone) feature a slower replacement with patient’s bone [36,37,38]. This seems to be related to a non-physiological recognition by the osteoclasts due to the absence of collagen and/or to physical alteration of the natural bone hydroxyapatite [3] due to the extremely high temperatures applied.

From a clinical point of view, several studies have shown that the anorganic bovine bone (ABB) integrates well with newly formed tissue and can be successfully used in different clinical applications, including ridge preservation, bone augmentation, and periodontal regeneration [7,8,14,15,16,23].

Recently, an anorganic equine bone (AEB) graft prepared by a high-temperature deproteinizing technique was introduced on the market. Since materials of equine origins may offer protection against iatrogenic prion disease transmission, there are no safety or ethical concerns regarding the use of AEB [39]. The purpose of this study concerns the determination of the morphological and physicochemical features of AEB, as well as its in vivo performance in an animal model of mandibular bony defects. As a control, the same types of analyses were performed on ABB, which has been present on the market for a long time, and its biophysical and clinical features are well documented in the literature [10,12].

The findings of the present study showed that AEB and ABB granules have an average diameter of 0.6–0.58 mm, with the majority of particles between 0.2 mm and 1.0 These data are in agreement with the data provided by the respective manufacturers (0.25–1 mm granule size). The electron microscopy images clearly identify the typical features of xenogeneic bone graft obtained by heat treatment. In particular, Figure 2 shows similar macropores and micropores in AEB and ABB, which may support new blood vessels colonization. The pore structure serves as physical scaffold for the migration of bone-forming cells [40,41]. Interestingly, it was previously reported that ABB exhibits a porosity of 70–75%, which promotes osteoconduction, enabling bone ingrowth into the inner part of the graft [19].

The crystallinity grade of AEB and ABB was assessed with XRD, which showed the typical peaks of hydroxyapatite [19]. To confirm the chemical composition of AEB, FT-IR analysis was performed, showing the bands associated with the chemical group of hydroxyapatite. The data analyzed showed the presence of phosphate ions at 1019 cm^−1^ and of the carbonate group at 1418 cm^−1^ and 875 cm^−1^, in agreement with the literature describing the heat treatment output of xenogeneic bone substitutes [42]. The same bands were detected for ABB. As expected, elemental chemical analysis through XRF revealed calcium and phosphorus as the main components of both AEB and ABB, with magnesium as a minor impurity. It is worth noting that both AEB and ABB showed a Ca/P ratio above 2, where the theoretical value of Ca/P ratio of hydroxyapatite is 1.67 [43]. This result is not surprising, as it is was already reported that an exchange of phosphate groups with carbonate groups can occur during the heating procedure [31]. This is shown by the carbonate peaks observed in the FT-IR spectra for both samples. A slight discrepancy emerged in the composition of trace elements between AEB and ABB. This is not surprising, as ion exchange can take place in the apatite component of the bone. Therefore, the composition of trace element varies considerably depending on some biological factors, such as nutrition and the turnover rate of the mineral [44].

Altogether, these findings confirm that the biophysical features of AEB are in agreement with that of the xenogeneic bone substitutes treated with high temperature and already described in literature [42]. Thus, one can expect a similar effectiveness in the repair of bone defects.

As proof of concept, the in vivo performance of AEB and ABB were evaluated in Yucatan mini-pigs. A randomized-controlled experimental study was conducted to measure the proportion of newly formed bone and remaining particles in standardized mandibular bony defects at 30 and 90 days after implantation. Even if the results must be considered as preliminary, due to the number of defects analyzed, a significant increase amount of newly formed bone was observed during time with both biomaterials. Considering the resorption rate, sites grafted with both materials showed a comparable progressive degradation pattern at the two time points considered. Local effects assessment showed a slight inflammatory and a minimal tissue response in all sites and at both time points, with no statistical differences between AEB and ABB, suggesting a neutral interaction of the grafted particles with the newly generated bone tissue.

This study, consistent with other works [37,45,46], confirmed that the use of ABB as a grafting material yielded a bone formation with no presence of inflammatory cell infiltrate. As the physicochemical structure affects the biological performance of the material [47,48], it is worthwhile considering that the similar manufacturing process could have an impact on the in vivo biomaterial behavior. In this respect, both materials exhibited a slow resorption rate as demonstrated by the observation of residual particles 90 days after surgery. In the literature there are several clinical studies showing a slower degradation of heat-treated xenogeneic bone substitutes, with the residual particles persisting in the grafted sites even years after biomaterial application [36,38,49]. As already noted, the interaction between osteoclasts and bone substitutes seems to be one central part of bone resorption. Solubility, microscopic structure, surface morphology, and physicochemical features have been proposed as regulators of osteoclastic adhesion and activity [3]. Based on these data, a better understanding of in vitro human osteoclasts behaviour when in contact with AEB would strengthen the correlation of the manufacturing process with the biological performance of the bone substitute.

## 5. Conclusions

In conclusion, the overall structural and physicochemical properties and pre-clinical evidence reported in the present study indicates that AEB has the typical features of heat-treated xenogeneic bone substitutes. Thus, it is reasonable to expect that AEB would yield similar results as other heat-treated xenogeneic bone substitutes in oral surgery procedures, and that it can be effectively used as bone grafting material. Further clinical studies are required to confirm this.

## Figures and Tables

**Table 1:** Abundance of the elements detected by XRF in the two samples. Limit of quantification (<LoQ) was 0.01 g/100 g.

Element	Abundance in AEB (g/100 g)	Abundance in ABB (g/100 g)
Aluminium	0.14	0.12
Barium	<LoQ	0.03
Calcium	34.95	34.99
Phosphorus	13.15	12.48
Magnesium	0.82	0.71
Strontium	0.04	0.04
Zinc	0.02	<LoQ
Sulphur	0.02	<LoQ

Not detected in both samples (<LoQ): antimony, silver, arsenic, bromine, cadmium, cobalt, chrome, iron, iodine, manganese, mercury, molybdenum, nickel, lead, potassium, copper, selenium, silicon, sodium, tin, thallium, tellurium, titanium, tungsten, vanadium, zirconium.

**Table 2:** Average histological scores of inflammatory data in AEB and ABB specimens.

*Time Points*	*AEB*	*ABB*
	*Polymorphonuclear cells*	
*30 days*	1.25 ± 0.5	1 ± 0
*90 days*	0.89 ± 0.3	0.66 ± 0.2
	*Lymphocytes*	
*30 days*	1 ± 0.5	0.75 ± 0.5
*90 days*	0.44 ± 0.5	0.5 ± 0.5
	*Macrophages*	
*30 days*	0.63 ± 0.5	0.25 ± 0.5
*90 days*	0.44 ± 0.5	0.17 ± 0.4
	*Plasma cells*	
*30 days*	0 ± 0	0.25 ± 0.25
*90 days*	0 ± 0	0 ± 0
	*Giant cells*	
*30 days*	0 ± 0	0 ± 0
*90 days*	0 ± 0	0 ± 0
	*Necrosis*	
*30 days*	0 ± 0	0 ± 0
*90 days*	0 ± 0	0 ± 0

Scores were assigned to each specimen on a scale of 0–5 according to ISO 10993-6:2007(E). Shown are the means ± SD. All comparisons between groups at 30 and 90 days are not significant (*p* > 0.05; Wilcoxon signed rank test with continuity correction).

**Table 3:** Average histological scores of soft tissue response in AEB and ABB specimens.

*Time Points*	*AEB*	*ABB*
	*Adipose tissue*	
*30 days*	0 ± 0	0 ± 0
*90 days*	0 ± 0	0 ± 0
	*Fibrosis*	
*30 days*	0.25 ± 0.70	0.5 ± 1
*90 days*	0 ± 0	0 ± 0
	*Neovascularization*	
*30 days*	1 ± 0	1.5 ± 0.6
*90 days*	1 ± 0	1 ± 0

Scores were assigned to each specimen on a scale of 0–5 according to ISO 10993-6:2007(E). Shown are the means ± SD. Adipose tissue, fibrosis, and neovascularization scores are not significantly different between groups at 30 and 90 days (*p* > 0.05; Wilcoxon signed rank test with continuity correction).

## Data Availability

Data sharing is not applicable for this article.

## Supplementary Materials

The following are available online at https://www.mdpi.com/article/10.3390/ma15031031/s1, Figure S1: Example of surgical pictures taken during the surgery: performing of surgical defects (A), grafting with the devices under investigation and protection of the grafting with pericardium membrane (B), suturing (C), opening of the surgical sites after specific time points (D), collection of bone samples (E), and storing of bone cores in 10% formalin (F).
