# Supplementary material for: A New Anorganic Equine Bone Substitute for Oral Surgery: Structural Characterization and Regenerative Potential
Source: Materials (Basel). 2022 Jan 28;15(3):1031. doi: 10.3390/ma15031031 (PMC8840601; doi:10.3390/ma15031031)
Supplement: Supplementary file 1 [file materials-15-01031-s001.zip › materials-1505141-supplementary.pdf]

Supplementary materials

A New Anorganic Equine Bone Substitute for Oral Surgery: Structural Characterization and Regenerative Potential

Alessandro Addis [1], Elena Canciani [2], Marino Campagnol [1], Matteo Colombo [3], Christian Frigerio [3], Daniele Recupero [3], Claudia Dellavia [2] and Marco Morroni [3,*]

[1] CRABCC Animal Lab, Biotechnology Research Center for Cardiothoracic Applications, Rivolta d'Adda, 26027 Cremona, Italy; alessandro.addis@crabcc.com (A.A.); marino.campagnol@unimi.it (M.C.)
[2] Department of Biomedical Surgical and Dental Sciences, Università Degli Studi di Milano, 20122 Milan, Italy; elena.canciani@unimi.it (E.C.); claudia.dellavia@unimi.it (C.D.)
[3] Bioteck S.p.A., Arcugnano, 36057 Vicenza, Italy; m.colombo@bioteck.com (M.C.); c.frigerio@bioteck.biz (C.F.); d.recupero@bioteck.biz (D.R.)
* Correspondence: m.morroni@bioteck.com
include country code; if there are multiple corresponding authors, add author initials)

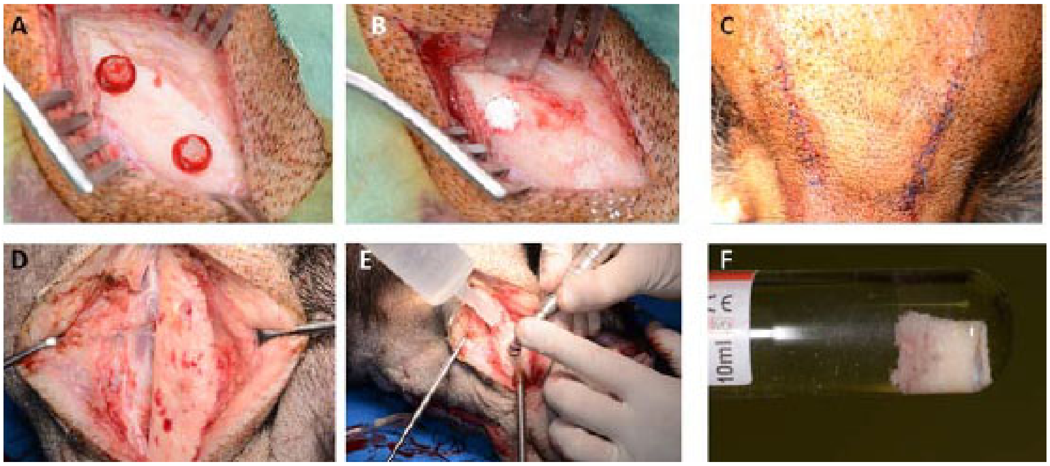

Figure S1. Example of surgical pictures taken during the surgery: performing of surgical defects (**A**), grafting with the devices under investigation and protection of the grafting with pericardium membrane (**B**), suturing (**C**), opening of the surgical sites after specific time points (**D**), collection of bone samples (**E**), and storing of bone cores in 10% formalin (**F**).
